# Acute Spontaneous Colonic Perforation in a Case of Newly Confirmed Scleroderma: Case Report

**DOI:** 10.2196/43295

**Published:** 2023-03-08

**Authors:** Glenn Goodwin, Christian Ryckeley, Davide Fox, Michael Ashley, Laurence Dubensky, Mauricio Danckers, Todd Slesinger

**Affiliations:** 1 Emergency Department Aventura Hospital and Medical Center Aventura, FL United States; 2 Department of Critical Care Aventura Hospital and Medical Center Aventura, FL United States; 3 Department of Radiology Aventura Hospital and Medical Center Aventura, FL United States

**Keywords:** scleroderma, systemic sclerosis, spontaneous bowel perforation, CREST syndrome, calcinosis, Raynaud phenomenon, esophageal dysmotility, sclerodactyly, and telangiectasis, multisystem connective tissue disorder, sclerosis, skin, dermatology, internal medicine, autoimmune, perforation, gastroenterology, esophagus, esophageal, connective tissue, emergency, gastrointestinal, case report

## Abstract

Scleroderma is a group of autoimmune diseases that principally affects the skin, blood vessels, muscles, and viscera. One of the more well-known subgroups of scleroderma is the limited cutaneous form of the multisystem connective tissue disorder known as CREST (calcinosis, Raynaud phenomenon, esophageal dysmotility, sclerodactyly, and telangiectasis) syndrome. In this report, we present a case of a spontaneous colonic bowel perforation in a patient with incomplete features of CREST. Our patient underwent a complicated hospital course involving broad-spectrum antibiotic coverage, surgical hemicolectomy, and immunosuppressives. She was eventually discharged home with a return to functional baseline status after esophageal dysmotility confirmation via manometry. Physicians managing patients with scleroderma ensuing to an emergency department encounter must anticipate the multitude of complications that can occur, as was seen in our patient. The threshold for pursuing imaging and additional tests, in addition to admission, should be relatively low, given the extremely high rates of complications and mortality. Early multidisciplinary involvement with infectious disease, rheumatology, surgery, and other respective specialties is crucial for patient outcome optimization.

## Introduction

### Background

Scleroderma, sometimes more colloquially known as systemic sclerosis, is a group of autoimmune diseases that principally affects the skin, blood vessels, muscles, and viscera [1]. One of the more well-known subgroups of scleroderma is the limited cutaneous form of the multisystem connective tissue disorder known as CREST (calcinosis, Raynaud phenomenon, esophageal dysmotility, sclerodactyly, and telangiectasis) syndrome [2]. Within the CREST syndrome spectrum of diseases exist numerous associated complications and conditions.

### Objective

The focus of this paper will be to present a case of a spontaneous colonic bowel perforation in a patient with incomplete features of CREST. Currently, only a few case reports exist documenting this relatively rare but recognized complication. Typically, gastrointestinal (GI) complications seen in CREST syndrome involve the esophagus; however, evolving data are demonstrating that concomitant distal GI pathologies are extremely common [3-6], as exemplified by this case.

### Ethical Considerations

We complied with all applicable laws and regulations concerning the privacy and security of patient personal information, including, but not limited to, the Health Insurance Portability and Accountability Act of 1996 and other US federal and state laws relating to the privacy and security of personally identifiable information. The patient provided her expressed and written consent for case report publication, with the family and coauthors present. Written consent was in accordance with the standardized hospital consent form. This case report was fully observational in nature and exempt from institutional review board approval.

## Case Report

A 60-year-old woman with a past medical history of chronic constipation, diverticulosis, vagus nerve cardiac pacemaker use, heavy tobacco use, and incomplete CREST syndrome presented to the emergency department (ED) for sudden onset of diffuse abdominal tenderness that began while watching television on the Ukrainian-Russian war. The patient was recently displaced from Ukraine and endorsed experiencing severe emotional stress, culminating in the abdominal pain episode that brought her into the ED. Additionally, due to the war, her medical records could not be obtained, and she only had a scant recollection of her previous medical conditions, limiting her definitive medical history. The patient had reportedly been diagnosed with one of the scleroderma spectrum of diseases approximately 10 years prior to presentation but had not been on any pharmacologic interventions for it thus far.

**Figure 1:**
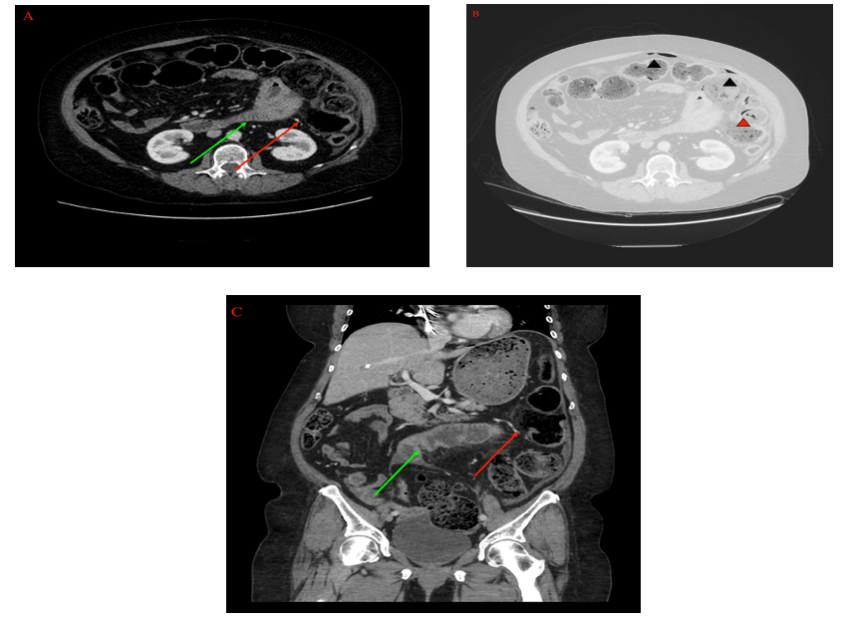
Systemic scleroderma presenting with spontaneous perforation: (A, B) axial and coronal images of contrast-enhanced computerized tomography at presentation demonstrating a localized perforation of the descending colon (solid arrows) with adjacent reactive small bowel wall thickening (green arrows); and (C) axial image in the lung window demonstrating the foci of free air (black arrowheads) in the anterior peritoneal cavity tracking from the perforated large bowel. Additional foci of pneumatosis coli are demonstrated in the adjacent descending colon (red arrowheads).

Upon arriving at the ED, the patient’s vital signs were significant for a blood pressure of 228/100 but otherwise normal with a pulse of 82 beats per minute, a respiratory rate of 14 breaths per minute, and an oxygen saturation level of 98% on room air. The patient’s physical examination was significant for a midline sternotomy scar from prior open heart surgery along with severe and diffuse abdominal tenderness. Serum laboratory values revealed an elevated lactic acid level of 4.0 mmol/L, along with a normal complete blood count, electrolytes, and coagulation values. The patient’s blood cultures subsequently grew *Bacteroides vulgatus*. The patient underwent a computed tomography scan of the abdomen and pelvis with intravenous contrast, which demonstrated a perforated descending colon along with adjacent intraperitoneal stool, free fluid and air, and pneumatosis (Figure 1). Other notable findings included moderate distention of the esophagus, likely secondary to reflux in addition to pneumobilia in the absence of a gallbladder.

The patient was immediately started on piperacillin-tazobactam in the ED followed by an emergent general surgery consultation. She underwent an exploratory laparotomy on the same day. The surgeons reported severe distention of the distal colon along with extensive adhesions. It was later revealed that the patient had undergone a previous exploratory laparotomy several years prior, which resulted in an open cholecystectomy and appendectomy.

The surgery concluded with lysis of adhesions, a partial left hemicolectomy and Hartman procedure, and end colostomy creation. The extensive GI findings precluded the complete closing of the initial midline entry incision, which was consequently left to heal by secondary closure. The patient was subsequently admitted to the intensive care unit (ICU).

While in the ICU, the patient experienced numerous complications including *Candida albicans* fungemia, *Bacteroides vulgatus* bacteremia, and exacerbation of her preexisting sclerodactyly as demonstrated by the worsening of her finger stiffness and swelling. The patient was noted to have persistently swollen laryngeal and pharyngeal tissues, making intubations extremely difficult. After a long and complicated ICU course, she was eventually extubated on postoperative day 10 and was transferred out of the ICU on postoperative day 13.

The patient’s blood infections were effectively treated with piperacillin-tazobactam, followed by meropenem and fluconazole, and then by micafungin. Serum laboratory values demonstrated complete resolution of all previously abnormal values.

While a biopsy is needed to definitively diagnose scleroderma, her scleroderma diagnosis was substantiated in the ICU with positive antinuclear and anticentromere antibodies along with an American College of Rheumatology/European League Against Rheumatism (ACR/EULAR) score of 15. The ACR/EULAR classification criteria are used to aid in the diagnosis of several rheumatological diseases, and scores of 9 or above in this validated scoring system are associated with scleroderma [6].

Additionally, the patient had baseline esophageal dysmotility, which was confirmed with manometry during her stay in the ICU. She was discharged home with outpatient rheumatology follow-up along with a daily steroid regimen soon after her transfer out of the ICU, with a general return to baseline functionality.

## Discussion

### Principal Findings

While up to 90% of patients with scleroderma are found to have some form of GI involvement, only 50% report symptom manifestation [7]. Notwithstanding that esophageal complications have been the most reported GI-related feature, colonic involvement is found almost as frequently, particularly in patients with abnormal esophageal manometry studies [3,7,8], as seen in our patient. Limited data exist relating to the correlation between the extent of disease and mortality; however, 1 study found that approximately 10% of deaths related to scleroderma were due to GI complications [9]. While many studies have demonstrated unfavorable patient outcomes relating to the lung and cardiac manifestations of the disease, GI involvement, particularly distal to the esophagus, portends poor survival [9,10]. While our patient’s definitive cardiac condition could not be determined, her history of open-heart surgery and use of the vagus nerve pacemaker may have been related to her scleroderma. Myocardial and vascular compromise has been well documented in patients with scleroderma, possibly accounting for the additional surprising feature of our patient being persistently hypertensive, even while septic [8].

Oropharyngeal dysphagia and deglutination abnormalities are found in up to 25% of patients with scleroderma [10]. Our patient’s exceptionally difficult airway may have reasonably been explained by these abnormalities. The patient was found to have extensive edema to the oropharynx and proximal larynx, refractory to high-dose steroids. Furthermore, the patient had repeated episodes of postextubation upper airway swelling, necessitating multiple reintubations.

In addition to the aforementioned complications, the patient also experienced several refractory and intractable infections. A possible explanation relates to findings from a study in 2022 by Kristofer et al [11], which explored the dysbiosis seen in many patients with scleroderma. The interplay between the hyperactive immune cells in the gut and the microbiome may have been responsible for many previously unexplained complications in patients with scleroderma [11]. Further compounding the interplay is the ubiquitous microvasculopathy and gut wall damage seen in scleroderma [8]. This patient’s extensive and prolonged hospital course could possibly be attributed to her underlying pathology, especially when considering the high mortality and morbidity associated with surgically repaired bowel perforations [12]. Bowel perforations that require surgery in otherwise healthy patients have been found to have an overall mortality rate of 10% to 15% and a morbidity rate of 20% to 30% [12].

### Conclusion

While much research exists regarding esophageal complications in patients with scleroderma, it is crucial for the clinician to consider the extremely high rates of extraesophageal GI involvement of the disease. This consideration may compel the emergency physician to have a lower threshold for additional testing and actions such as cross-sectional imaging, lab tests, and specialist consultations. This recommendation is particularly germane for patients who are not being treated for their disease, as was the case with our patient. Additionally, physicians managing patients with scleroderma ensuing to an ED encounter must anticipate the multitude of complications that can occur. Preemptively involving surgical and anesthesia teams early in the inpatient course is both reasonable and appropriate. Additionally, early multidisciplinary inpatient involvement with infectious disease and rheumatology is crucial for outcome optimization. While the focus of this paper was on the relatively rare complication of spontaneous intestinal perforation, the secondary objective was to illuminate the possibility of many others.

## Abbreviations

ACR/EULAR: American College of Rheumatology/European League Against Rheumatism
CREST: calcinosis, Raynaud phenomenon, esophageal dysmotility, sclerodactyly, and telangiectasis
ED: emergency department
GI: gastrointestinal
ICU: intensive care unit
